# Effects of Non-Conventional Sterilisation Methods on PBO-Reinforced PVA Hydrogels for Cartilage Replacement

**DOI:** 10.3390/gels8100640

**Published:** 2022-10-09

**Authors:** Tomás Pires, Andreia Sofia Oliveira, Ana Clara Marques, Madalena Salema-Oom, Célio G. Figueiredo-Pina, Diana Silva, Ana Paula Serro

**Affiliations:** 1 Centro de Química Estrutural (CQE), Institute of Molecular Sciences, Departamento de Engenharia Química, Instituto Superior Técnico, Universidade de Lisboa, Av. Rovisco Pais, 1049-001 Lisbon, Portugal; 2 Instituto de Engenharia Mecânica (IDMEC), Instituto Superior Técnico, Universidade de Lisboa, Av. Rovisco Pais 1, 1049-001 Lisbon, Portugal; 3 Centro de Investigação Interdisciplinar Egas Moniz (CiiEM), Instituto Universitário Egas Moniz, Quinta da Granja, Monte da Caparica, 2829-511 Caparica, Portugal; 4 CERENA, DEQ, Instituto Superior Técnico, Universidade de Lisboa, Avenida Rovisco Pais, 1049-001 Lisboa, Portugal; 5 CDP2T, Escola Superior de Tecnologia de Setúbal, Instituto Politécnico de Setúbal, 2910-761 Setúbal, Portugal; 6 CeFEMA, Instituto Superior Técnico, Universidade de Lisboa, 1049-001 Lisbon, Portugal

**Keywords:** articular cartilage substitutes, PVA hydrogels, PBO nanofibres, sterilisation, microwave, high hydrostatic pressure, plasma

## Abstract

Articular cartilage (AC) degradation is a recurrent pathology that affects millions of people worldwide. Polyvinyl alcohol (PVA) hydrogels have been widely explored for AC replacement. However, their mechanical performance is generally inadequate, and these materials need to be reinforced. Moreover, to be used in a clinical setting, such materials must undergo effective sterilisation. In this work, a PVA hydrogel reinforced with poly(p-phenylene-2,6-benzobisoxazole) (PBO) nanofibres was submitted to three non-conventional sterilisation methods: microwave (MW), high hydrostatic pressure (HHP), and plasma (PM), in order to evaluate their impact on the properties of the material. Sterilisation was achieved in all cases. Properties such as water content and hydrophilicity were not affected. FTIR analysis indicated some changes in crystallinity and/or crosslinking in all cases. MW was revealed to be the most suitable method, since, unlike to PM and HHP, it led to a general improvement of the materials’ properties: increasing the hardness, stiffness (both in tensile and compression), and shear modulus, and also leading to a decrease in the coefficient of friction against porcine cartilage. Furthermore, the samples remained non-irritant and non-cytotoxic. Moreover, this method allows terminal sterilisation in a short time (3 min) and using accessible equipment.

## 1. Introduction

Osteoarthritis is a degenerative joint disease that affects more than 500 million people worldwide and is recognized as the main cause of permanent disability. Rheumatoid arthritis, injuries, or overuse can also lead to joint damage. The hip and knee are the most commonly affected joints, due to the large loads sustained [1]. Articular cartilage (AC) is a highly hydrated connective tissue that covers the ends of bones in diarthrodial joints. It consists of a porous elastic solid matrix, containing interstitial fluid, and exhibits high load-bearing and low-friction capabilities. Such features arise from its biphasic composite structure, in conjunction with the lubrication characteristics of the synovial fluid (SF) [2,3]. The capability of cartilage to repair and regenerate is limited, due to its aneural and avascular nature [4,5,6]. As such, the progression of cartilage degradation is generally inevitable.

Presently, the available treatment options for injured AC range from physiotherapy and/or the use of anti-inflammatories or viscosupplementation, when the damage is mild, to total joint arthroplasty, in the more severe cases [7,8]. Since the latter is a quite invasive procedure, new treatment options have been pursued, to manage the problem in earlier stages [9]. Cartilage replacement, by filling the defector in the form of a hemiarthroplasty counter face, has been considered, as well as the possibility of using an interposition device to alleviate pain and/or correct joint deformity [10]. Regardless, replicating the unique biphasic and heterogeneous structure of AC is a challenging task. Among the studied materials, hydrogels have emerged as a promising alternative, owing to their high-water contents, porous structure, and viscoelastic mechanical properties that resemble those of cartilage tissue [11,12].

Polyvinyl alcohol (PVA) has received special attention due to its excellent biocompatibility and non-toxicity. However, simple PVA hydrogels usually present insufficient mechanical properties to resist the high loads experienced by diarthrodial joints [13]. The preparation procedure may critically affect the performance of these materials. Cast-drying and freeze-thawing are the most commonly used methods to obtain these hydrogels. In a previous work [14], the authors found that PVA hydrogels obtained by cast-drying better mimic the natural cartilage tissue, in terms of morphology, water content, and tribomechanical properties. However, an improvement of these properties was still required. Different strategies have been used for this purpose, e.g., post-production physical treatments, such as thermal annealing [10,15], or the incorporation of reinforcement agents into the hydrogel matrix, such as nanofibres or nanoparticles [16,17,18,19]. The addition of nano/microfibres to a PVA hydrogel polymer network represents a promising method for cartilage substitution, since this can mimic the collagen fibres present in the extracellular matrix of natural cartilage.

In a recent work, Oliveira et al. [20] synthesized a PVA hydrogel reinforced with poly(p-phenylene-2,6-benzobisoxazole (PBO, commercial name Zylon^®^), which demonstrated excellent mechanical properties for cartilage substitution, when the damage is still circumscribed. In particular, it presented a large water content, high mechanical stiffness, low friction, and excellent biocompatibility. PBO fibres show an extremely high tensile strength (~5.8 GPa), approximately 1.6-times higher than Kevlar^®^. Its tensile elastic modulus depends on the type of fibre, ranging from 180 or 270 GPa for “as spun” (AS) or “high modulus” (HM) fibres, respectively. PBO’s compressive strength is significantly lower than its tensile strength, falling between 0.469 and 0.561 GPa. PBO also shows high thermal stability (up to 650 °C) [21]. The PBO fibres can be tailored into nanofibres through acid-catalysed hydrolysis. The high aspect ratio of the nanofibres can lead to multiple fibre interconnections, promoting gelation. Furthermore, this process creates functional sites in the nanofibres (i.e., carboxyl, amino and phenolic hydroxyl groups), which can be combined with PVA molecules through hydrogen bonding [20,22].

Sterilisation is a mandatory step for biomaterials intended for AC replacement, in order to minimize the incidence of medical device-related infections. Terminal sterilisation methods can induce changes to several material properties (e.g., aspect, size, colour, chemical structure, physical integrity, and biocompatibility). Many hydrogels are sensitive to conventional sterilisation methods (e.g., steam and heat or gamma-radiation) [23]. The presence of water in these materials makes their sterilisation even more challenging, as the water molecules may have harmful effects, such as the breaking of chemical bonds [24]. Thus, non-conventional sterilisation methods have been explored, to overcome possible detrimental damage to biomaterials during processing.

Microwave irradiation (MW) is an accessible method that relies on the exposition of the materials to electromagnetic radiation in the microwave frequency range (300 MHz and 300 GHz). This induces rotation of the polar molecules (as water) and ionic polarization, leading to a rapid increase in temperature [25], which results in the irreversible denaturation of enzymes and structural proteins essential for microorganisms’ viability and reproduction [26]. Another method recently investigated for the sterilisation of hydrogels is high hydrostatic pressure (HHP) [27,28]. In this method, the packaged materials are immersed in a water bath and submitted to high pressures (e.g., 100–1000 MPa) at relatively low temperatures (50–70 °C). The high applied pressures induces changes in the microorganisms’ cell membranes that lead to the leakage of the intracellular contents and also denature key enzymes [29]. Low-temperature glow discharge plasma treatment has also been investigated for its potential to sterilise polymeric materials. Gas plasma (PM) is created through the application of strong electromagnetic fields in an inert gas atmosphere, forming free radicals that cause etching of the cells’ surfaces, leading to the release of the inner contents from living cells [30,31].

This work aimed to study the effect of these three non-conventional sterilisation methods (i.e., MW, HHP, and PM) on a PBO nanofibre reinforced PVA hydrogel intended to be used as cartilage replacement material. The efficacy of sterilisation was accessed, and the sterilised hydrogels were characterised in terms of their structural and mechanical properties, wettability, swelling capacity, and rheological behaviour, to evaluate possible effects of the different sterilisation procedures. Finally, the biocompatibility of the hydrogels was ascertained via irritability (Hen’s egg test–chorioallantoic membrane, HET–CAM test) and cytotoxicity assays.

## 2. Results and Discussion

PVA has been, to date, the most investigated synthetic hydrogel for cartilage replacement. However, PVA alone presents mechanical and tribological properties below those of natural cartilage [32]. Therefore, the reinforcement of PVA with fibres, and/or co-polymerization with other compounds, has been attempted, to improve its properties [33]. In this work, PVA/PBO hydrogels were produced through the cast-drying method, which leads to the formation of hydrogen bonds between PVA chains [34] and of those of PBO [20]. The PBO fibres were solubilized in a TFA (trifluoroacetic acid)/MSA (methanesulfonic acid) mixture, to carry out acid-catalysed hydrolysis, tailoring them into nanofibres.

Sterilisation is a mandatory step, to ensure the biological safety of cartilage replacement materials. However, it can change their properties and impair their performance in extreme cases. Conventional sterilisation methods often rely on exposure to heat and highly energetic radiation (e.g., steam heat, gamma irradiation), but PVA hydrogels are generally sensitive to such agents [35]. In this sense, this work aimed to evaluate the effect of non-conventional sterilisation methods on PVA/PBO hydrogels, to identify a possible alternative that, while being effective, does not harm them. The materials were exposed to microwaves, HHP, and Argon (Ar) plasma, and the efficacy of sterilisation was verified. In all cases, they were processed in the hydrated state, except for plasma, where samples were first dried. Such a procedure was adopted, as the presence of water, even in small amounts, makes the argon plasma unstable and dangerously increases the temperature (which may reach temperatures of several hundreds of °C) [36], potentially damaging the hydrogels. Therefore, plasma-treated samples require a posterior hydration, which must be performed in aseptic conditions.

### 2.1. Microbiological Load and Sterility Assessment

Determination of the microbiological load of the non-sterile PVA/PBO samples showed a low bioburden value, in the order of 10^2^ CFU/sample for bacteria, and of 7.4 ± 2.8 CFU/sample for fungi. This was expected, taking into account that the method of synthesis of the PVA/PBO hydrogels involved highly acid solutions. Nevertheless, the potential for infection with such a bioburden is still of high risk.

**Figure 1:**
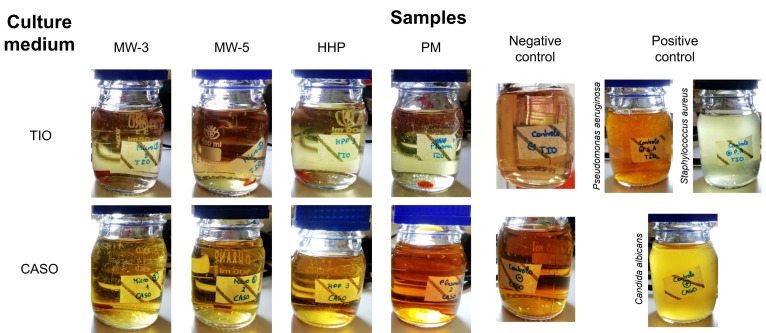
Culture media for bacteria and fungi after 14 days of incubation for MW-3, MW-5, HHP, and PM PVA/PBO hydrogel samples. The negative and positive controls are also shown.

The sterility tests did not allow visualizing any signs of contamination in the culture media for all sterilisation processes after the 14 days of incubation (Figure 1), demonstrating that all the studied methods ensured biologically safe materials. Exposure to plasma for 3 or 5 min (MW-3 and MW-5, respectively), led to sterile materials in both cases, whereby only MW-3 samples were kept in the study.

### 2.2. Morphology

**Figure 2:**
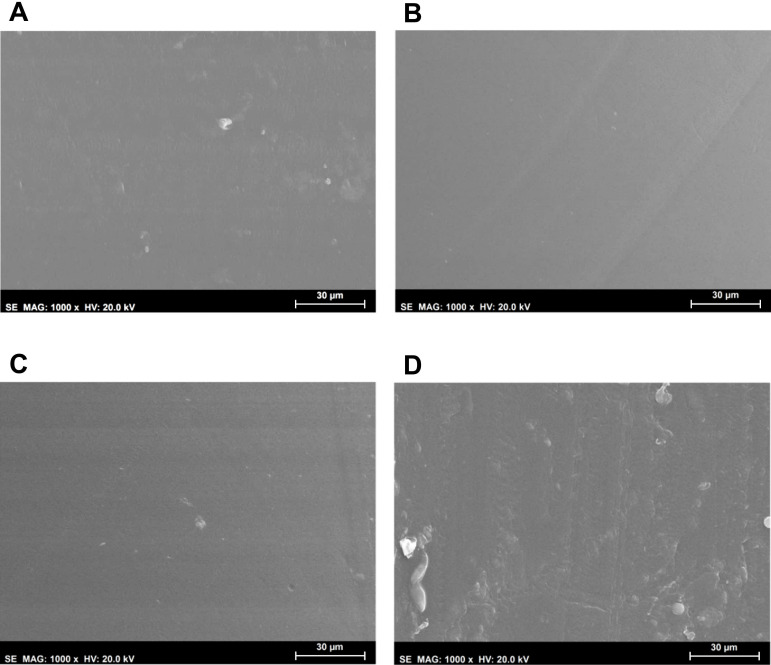
SEM micrographs (1000× magnification, scale bar 30 μm) of the PVA/PBO hydrogels: non-sterile (**A**), MW-3 (**B**), HHP (**C**), and PM (**D**) samples.

Morphological analysis of the hydrogels’ surfaces by SEM (Figure 2) showed some irregularities on non-sterile samples, which had almost disappeared after HHP treatment. MW led to an even smoother surface. This effect may have resulted from the temperature to which the samples were submitted during the sterilisation procedures, and in the case of HHP, also from the high pressure: 70 °C, 600 MPa for 10 min for HHP; and 100 °C, atmospheric pressure for almost 3 min for MW. In contrast, the plasma induced some heterogeneity, leading to the appearance of protuberances and an increase in the surface’s roughness. Moreover, etching-derived holes could also be observed in these samples. Etching is a common side effect of the plasma procedure, being primarily caused by the enhanced energies of the ions and radicals that strike the surfaces [30,37].

### 2.3. Chemical Structure

**Figure 3:**
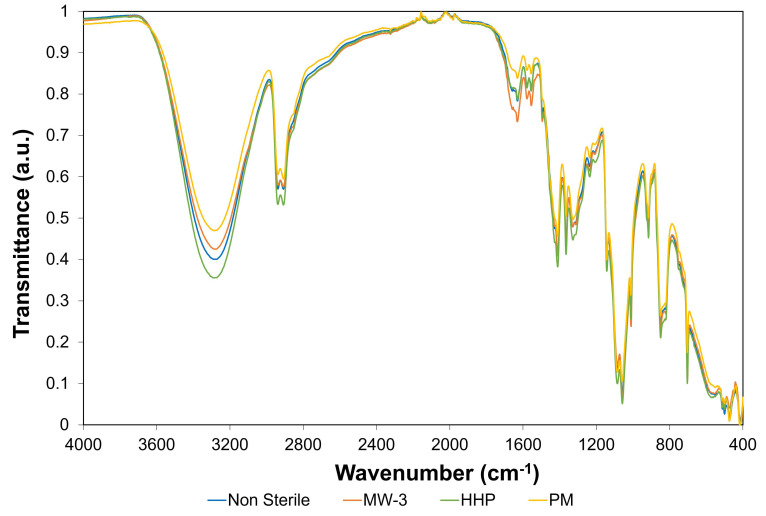
FTIR-ATR spectra of the non-sterile and sterile PVA/PBO hydrogels, in the region of 4000–400 cm^−1^.

Fourier-transform infrared spectroscopy (FTIR) analysis was carried out on the non-sterile and sterile hydrogels, to attain information regarding possible chemical modifications induced by the sterilisation procedures. Figure 3 shows the FTIR spectra of the different hydrogel types.

The hydrogels’ spectra demonstrated several similarities to the PVA spectrum observed in a previous study [20]. This was expected, since PVA had a much higher proportion in the hydrogel composition. Additionally, no new peaks arose from the sterilisation procedures.

The large band observed at ca. 3280 cm^−1^ is linked to the stretching of O–H bonds. The vibrational bands observed between 3000 and 2840 cm^−1^ refer to alkyl group C–H bonds stretching, and that at ca. 1085 cm^−1^ to C-O-C, i.e., C-O in secondary alcohols, such as those typical of PVA structures [17,38]. The region between 1700 and 1500 cm^−1^ exhibits peaks typically attributed to the stretching of C-C bonds and the covalent double bonds C=N and C=C from PBO, and the C=O present in PVA [20,39]. The most significant difference was the higher intensity of the peaks within the region 1700–1500 cm^−1^ for MW-3 samples, compared to non-sterilised ones. This is in agreement with what was observed in other studies regarding the effect of microwaves on PVA and PVA/graphene nanocomposites [40]. These authors found an increase in the C=O and C=C peak intensity after 3 min irradiation at 200 W and hypothesized that microwaves led to side chain scission, enhancing the subsequent bonds between the polymer chains, i.e., the formation of a more cross-linked structure. At lower wavenumbers, but above 1300 cm^−1^, several peaks were identified, corresponding to C-N stretching of PBO and CH_2_ bending of PVA, while the peak at 704 cm^−1^ was ascribed to the C-H bending of PBO [39].

According to the literature [41], FTIR allows inferring the degree of crystallinity of hydrogels through the analysis of the peak at 1141 cm^−1^. The intensity of this peak is influenced by the crystalline portion of the polymeric chains, which is related to the symmetric C–C stretching mode or stretching of the C–O of a portion of the chain, where an intramolecular hydrogen bond is formed between two neighbouring OH groups that are on the same side of the plane as the carbon chain. Following a procedure similar to that adopted in [41], the ratio of band intensities (height) of the 1141 cm^−1^ (C-O crystallinity) and 1085 cm^−1^ band (C-O-C bond stretching in PVA structure, secondary alcohol), was calculated for each sample (Table 1). It should be noted that this latter band did not significantly vary with the treatments. This analysis revealed that samples MW-3 and HHP were the ones exhibiting a higher crystallinity, followed by the PM sample. In fact, the highest peak ratio value observed for HHP was likely be due to the extremely high pressures occurring during the HHP process, which might decrease the distance between the polymeric chains, inducing the rupture of some intra/inter-molecular bridges and/or the formation of new bonds, namely hydrogen bonds [42,43], and favouring order at long distance, i.e., the formation of crystalline regions.

Regarding the O-H stretching vibration peak (ca. 3280 cm^−1^), although no significant change in its wavenumber was observed, a decrease in terms of intensity was found for the plasma samples (PM), as Table 1 reveals through the peak ratio of the OH bonding at 3280 cm^−1^ and that of the C-O bond at 1085 cm^−1^ (I_3280_ cm^−1^/I_1085_ cm^−1^). The effect of plasma on the materials strongly depended on the conditions used (e.g., power, time, gas flux). The literature is not consensual on the influence of argon plasma on PVA-based materials: while some authors [44] found significant changes in the FTIR spectra, such as the appearance of new peaks associated with amide, carboxylic acid, and OH/NH functionalities, others [45] reported minor changes.

Concerning the samples MW-3 and HHP, a slight increase in the ratio I_3280_ cm^−1^/I_1085_ cm^−1^ was observed relative to the non-sterile samples. The higher degree of crystallinity of these samples (referred above) is probably associated with the higher number of OH groups, important for establishing H bonds.

Finally, Table 1 shows a decrease for the PM samples in the relative intensity of the peak at 1056 cm^−1^, ascribed to C-O bond stretching in primary alcohols, when compared to that of C-O bond stretching in the secondary alcohols typical of the PVA macromolecular chains (I_1056_ cm^−1^/I_1085_ cm^−1^). This is in line with the lower amount of OH groups found for the PM samples, i.e., a lower peak ratio I_3280_ cm^−1^/I_1085_ cm^−1^. In turn, the MW-3 samples exhibited a higher value for this peak ratio, suggesting more primary alcohol moieties than the non-sterilised samples, which might eventually indicate some chain breaking.

### 2.4. Water Content and Wettability

**Figure 4:**
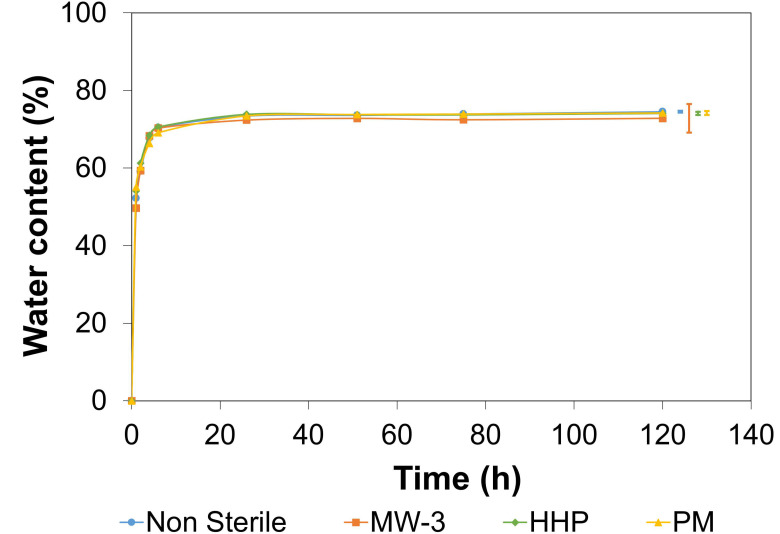
Water content over time for non-sterile and sterile PVA/PBO hydrogels. The error bars represent the ± mean standard deviations (*n* = 3).

The hydration process kinetics were studied over time (Figure 4). No significant differences were observed between the sterile and non-sterile materials (*p* = 0.8793). A sharp rise in *WC* was observed in the first 5 h, with equilibrium being reached after 24 h (values in the range 73–74.5%). Such values fall within the range observed for AC (65–80%) [46].

The wettability of polymers is often defined as a crucial parameter for cell attachment [47]. The optimal contact angle values seem to depend on the type of cells, but research suggests that moderate values are more desirable than having hydrogels with a very high or low wettability [48]. The untreated PVA/PBO hydrogels led to an average contact angle value of 40 ± 3° (in the range of those found in literature [15,49]), showing an hydrophilic behaviour. The sterilisation methods did not cause meaningful modifications of the samples’ wettability (MW-3 39 ± 3°, HHP 39 ± 4°, PM 40 ± 4°).

### 2.5. Mechanical Behaviour

The mechanical properties of cartilage replacement materials are critical, since in the body they will experience a variety of mechanical forces, namely compression, tension, and shear [50]. In order to minimize strain mismatch at the implant–tissue interface, the materials must present properties as similar as possible to the natural tissue.

#### 2.5.1. Compressive Tests

**Figure 5:**
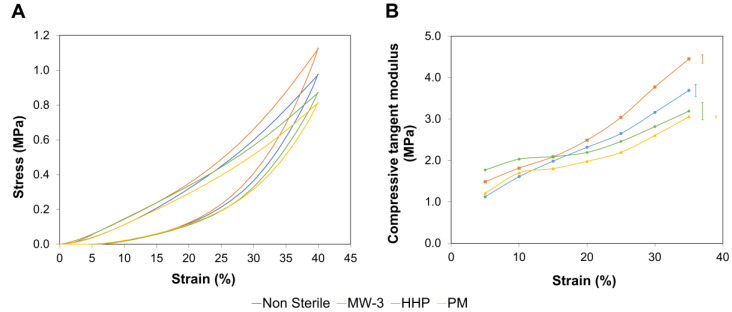
Typical compressive stress-strain curves (**A**) and compressive tangent modulus (**B**) for non-sterile and sterile PVA/PBO hydrogels. The error bars represent the mean ±standard deviations (*n* = 3).

Figure 5A shows typical compressive stress-strain curves obtained for the PVA/PBO hydrogels.

Since hydrogels are biphasic materials, in the initial stages of the compression experiments, the applied load was essentially supported by the liquid contained in the hydrogels. Raising the load leads to the exudation of the fluid from the matrix, being the load transferred to the solid phase. This is reflected by an increase in the slope of the curves.

Figure 5B depicts the compressive tangent modulus between 5 and 35% strains. Although all samples showed an increase of this parameter over the entire strain range tested, the rate of increase was different for the various tested sterilisation treatments. This leads to distinct compressive behaviours, depending on the compressive strain. Bellow 15%, the HHP and MW-3 samples exhibited a higher tangent compressive modulus, which might have been due to the higher crystallinity level of these samples, as detected by FTIR analysis. Above this strain, the MW-3 samples stood out, with superior values, which may have resulted from the higher content of intramolecular covalent double bonds.

#### 2.5.2. Tensile Tests

**Figure 6:**
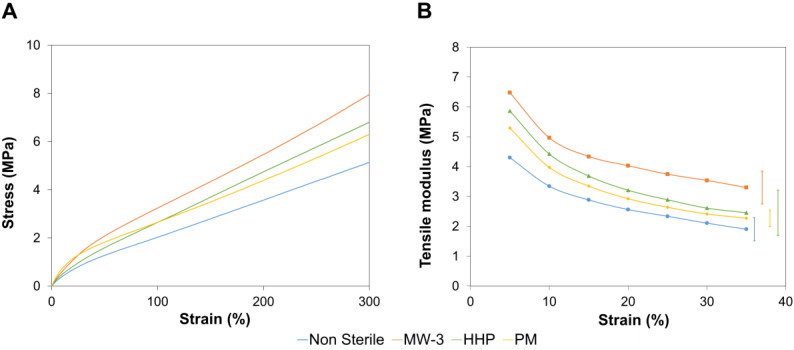
Typical tensile stress-strain curves (**A**), and tangent tensile modulus (**B**) for non-sterilised and sterilised PVA/PBO hydrogels. The error bars represent the mean ±standard deviations (*n* = 3).

Representative tensile stress-strain curves of all tested samples are shown in Figure 6, as well as their tensile tangent modulus. All the sterilisation treatments led to an increase in the tangent tensile modulus, due to the higher crystallinity level, as concluded from the FTIR spectra. The highest value was observed for MW-3 samples, possibly due to the greater content of covalent double bonds C=N and C=C from PBO, and the C=O present in PVA. It should be emphasized that, contrarily to the compressive tangent modulus, the tensile modulus decreased with strain. Indeed, both compressive and tensile stresses promoted, in the initial stage, the release of water from the hydrogels. However, as the stress was increased, compression led to the interpenetration of the polymer chains, resulting in the materials’ hardening, while stretching may cause the rupture of hydrogen bonds (that ensure the intermolecular physical crosslinking), allowing the slipping of the chains over each other and resulting in a lower material stiffness [15].

#### 2.5.3. Rheological Tests

**Figure 7:**
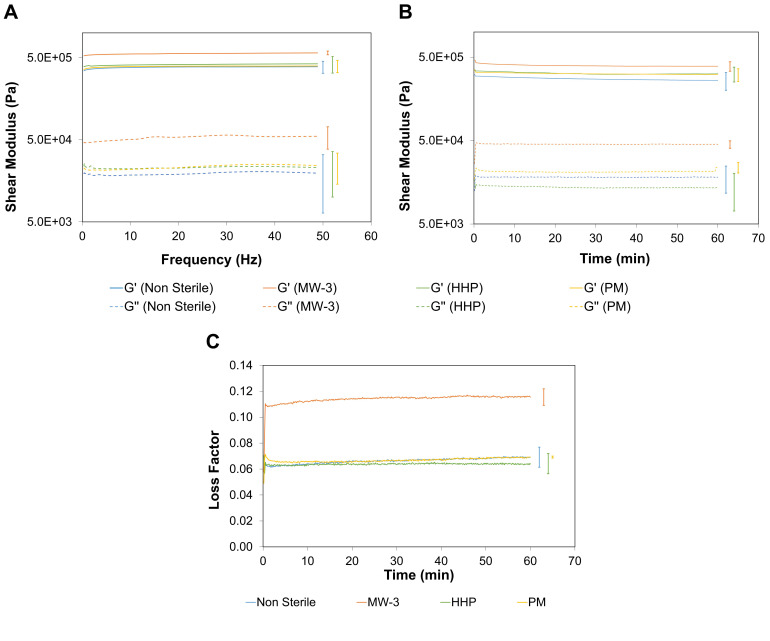
Storage (G′) and loss (G″) moduli for non-sterile and sterile PVA/PBO hydrogels (**A**) as a function of the frequency (0.1–50 Hz) and (**B**) of time (1 h) with a frequency of 1 Hz, both at a fixed strain rate γ = 0.1%. Loss factor variation with time (**C**). The error bars are the ± standard deviation (*n* = 3).

Oscillatory shear mode testing was conducted, by carrying out a frequency sweep analysis in the range of 0.1–50 Hz within the LVER at a fixed shear strain of γ = 0.1% (determined through an amplitude sweep assay). Figure 7A shows that for all hydrogels, *G′* was about one order of magnitude higher than *G″*, which indicates that, independently of having been sterilised or not, they mainly exhibited a solid elastic behaviour, within the frequency range studied. Measurements of *G′* and *G″* at a fixed strain (γ = 0.1%) and frequency (ω = 1 Hz) were carried out as a function of time for 1 h (Figure 7B). Again *G′* > *G″* for all hydrogels. Microwave treatment resulted in an increase of both parameters, but this was more accentuated for *G′*, showing that after this treatment the material could store a higher amount of energy. These samples showed a larger loss factor (Figure 7C), and therefore a higher damping capacity, which may have resulted from the larger amount of primary alcohol moieties, suggesting the presence of smaller segments embedded within the long polymeric PVA chains.

It should be underlined that both the non-sterile and sterile PVA/PBO hydrogels showed a shear modulus value (G* = (*G′*^2^ + *G″*^2^)^1/2^) within the reported range for AC. In fact, although the reported values for the G* of AC vary between studies, they are typically in a range of 0.2–2.5 MPa [51]. Studies of lamb cartilage plugs on a similar device as the one used in this study (1 Hz frequency and effective shear strain amplitude of 0.023%) reported a storage moduli in the range of 0.4–0.6 MPa [52].

#### 2.5.4. Hardness Tests

**Figure 8:**
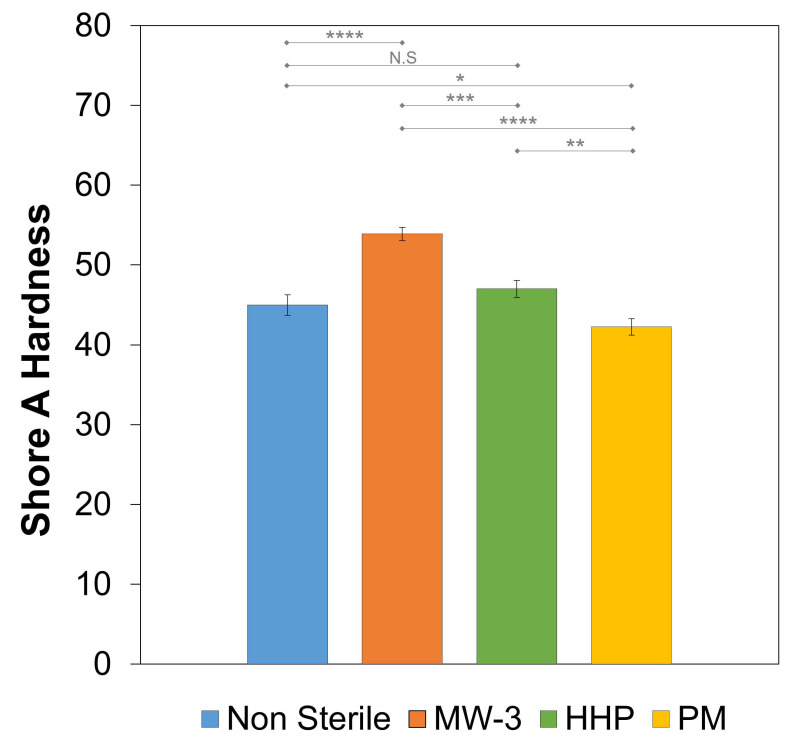
Shore A hardness values for the non-sterile and sterile PVA/PBO hydrogels. The error bars are the ± standard deviation (*n* = 3). Statistical analysis was performed using Student-*t* test, with significance set at * *p* < 0.05, ** *p* < 0.01, *** *p* < 0.005, **** *p* < 0.001. N.S. = not significant.

The Shore A hardness value of the non-sterile hydrogel was 45.0 ± 1.3 (Figure 8). HHP treatment did not led to a statistically significant variation of this property (*p* = 0.1102), while plasma treatment slightly affected it (*p* = 0.0191). However, MW-3 led to an increase of ≈18 (*p* < 0.001). This is in agreement with the superior mechanical behaviour observed for these samples and may have been a result of the higher crystallinity and, possibly, higher degree of cross-linking.

### 2.6. Tribological Behaviour

**Figure 9:**
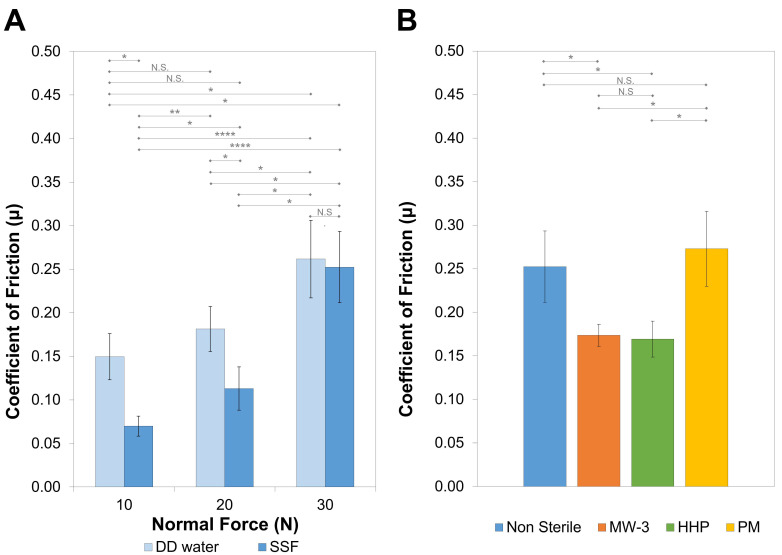
Coefficient of friction values for the non-sterile PVA/PBO hydrogels at different applied loads, using DD water and SSF as lubricants (**A**), and of the non-sterile and sterile hydrogels with 30 N of normal load and SSF as lubricant (**B**). Porcine cartilage was used as the counter body. The error bars represent the mean ± standard deviations (*n* = 3). Statistical analysis was performed using a Student-*t* test, with significance set at * *p* < 0.05, ** *p* < 0.01, **** *p* < 0.001. N.S. = not significant.

The coefficient of friction of the non-sterile hydrogel was measured against porcine cartilage samples, using a normal load of 10, 20, and 30 N. Porcine cartilage is significantly similar to human cartilage, and therefore is often used as a model to study the effects of friction between cartilage surfaces and synthetic materials. DD water and SSF were used as lubricants, the latter to better simulate the pseudo-synovial fluid. The average values obtained after stabilization of the coefficient of friction are given in Figure 9A. They increased with the normal load (*p* = 0.0056 and *p* = 0.0035 for DD water and SSF, respectively). This was expected, due to the higher deformation of the hydrogel, which should increase the contact area between the sliding surfaces and, therefore, the adhesion forces. In addition, lower coefficient of friction values were observed with SSF lubricant. The presence of hyaluronic acid and albumin significantly increased the viscosity of the fluid (17.5 ± 0.7 mPa.s compared to ≈1 mPa.s for water at room temperature), enhancing the hydrodynamic component of the lubrication. This contributed to a mixed lubrication regime over the boundary the one that should occur with DD water [53]. Moreover, the adsorption of the biomolecules on the surfaces gives rise to a protective layer that will contribute to decreasing the friction between the surfaces.

The effect of the sterilisation procedures was only studied for the 30 N load (average contact pressure ≈1 MPa) and using SSF as lubricant (Figure 9B). MW-3 and HHP samples led to coefficients of friction about 30% lower than the non-sterile hydrogels (*p* = 0.0406 and *p* = 0.0412, respectively), while PM samples showed a slight increase (≈8%), but this was not statistically significant. The coefficient of friction was affected by multiple factors, for example, the surfaces’ adhesion, deformation, and roughness. In addition, in the presence of biological molecules, this also depends on the characteristics of the adsorbed layer (rigid or viscoelastic), which is determined by the surface chemical structure, polarity, and roughness. Although the interpretation of the friction results was not straightforward, they fell within the range of values found in the literature for natural cartilage, which range from 0.002 to 0.5 [54], depending not only on the experimental conditions used (e.g., test configuration, lubricant, applied load), but also on the type of cartilage and donor age [7,8].

**Figure 10:**
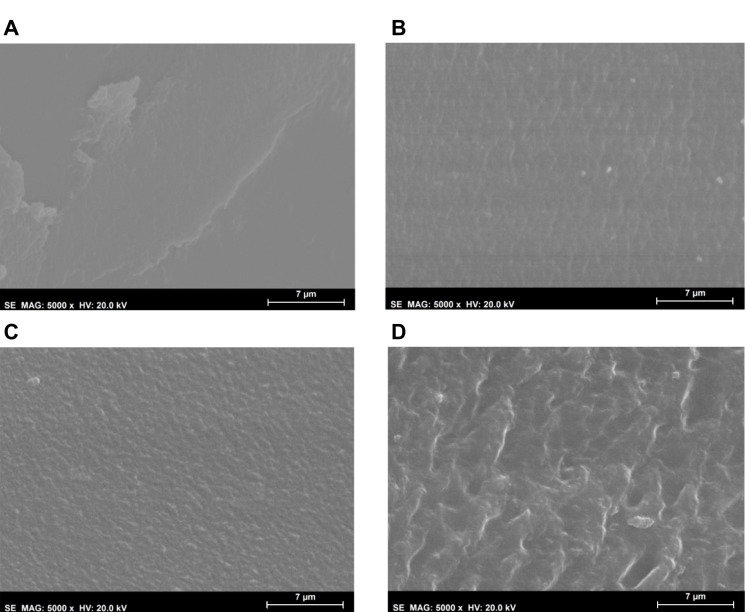
SEM micrographs (5000× magnification, scale bar 7 μm) of the PVA/PBO hydrogels inside the wear tracks, produced in the tribological experiments. Non-sterile (**A**), MW-3 (**B**), HHP (**C**), and PM (**D**) samples.

After the tribological tests, marks were clearly visible on the hydrogels’ surface. SEM images of the inside of the wear tracks caused by the tribological tests performed with an applied load of 30 N in SSF are depicted in Figure 10. The non-sterile samples showed some signs of delamination. MW-3 and HHP treatments induced some rippling on the surfaces, which become rougher. According to Dong et al. [55], ripples may be formed due to a stick-slip process that occurs when the hydrogels are pulled and deformed along the sliding direction by the sliding counter-body. This effect was more obvious for the PM samples, suggesting that adhesion was more prominent for this material, which is in agreement with the higher values observed for the coefficient of friction.

### 2.7. HET-CAM Test

The HET-CAM test is a well-established prediction model for tissue irritation due to chemicals, based on the observation of signs of lysis, haemorrhage, or coagulation in the chorioallantoic membrane (CAM) surrounding the chicken embryo in eggs. Although cartilage presents an avascular structure, the surrounding tissues of the joints and bone are vascularized, thereby making the HET-CAM test relevant.

**Figure 11:**
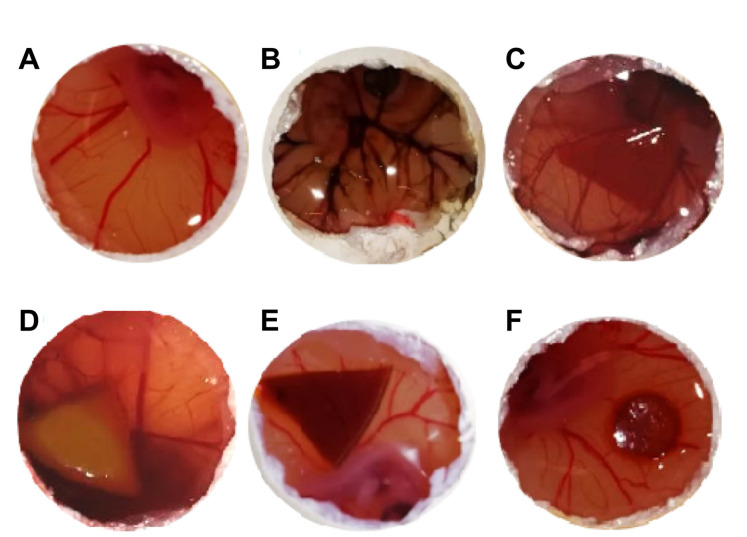
Chorioallantoic membrane images after 5 min of exposure to the negative control (NaCl 0.9%) (**A**), positive control (NaOH, 1 M) (**B**), and to the non-sterile (**C**), MW-3 (**D**), HHP (**E**), and PM (**F**) hydrogel samples.

Photographs of the CAM after 5 min of direct contact with the non-sterile and sterile PVA/PBO hydrogels are presented in Figure 11, as well as the positive control (after the addition of NaOH) and the negative control (after the addition of NaCl solution). In the positive control, visible signs of severe irritation can be observed (Figure 11F). Both sterilised and non-sterilised hydrogels induced a behaviour similar to the one observed for the negative control (Figure 11E), i.e., no visual signs of lysis, haemorrhage, or coagulation, were observed. Therefore, the IS was equal to 0, and the hydrogels could be classified as non-irritant.

### 2.8. Cytotoxicity Analysis

**Figure 12:**
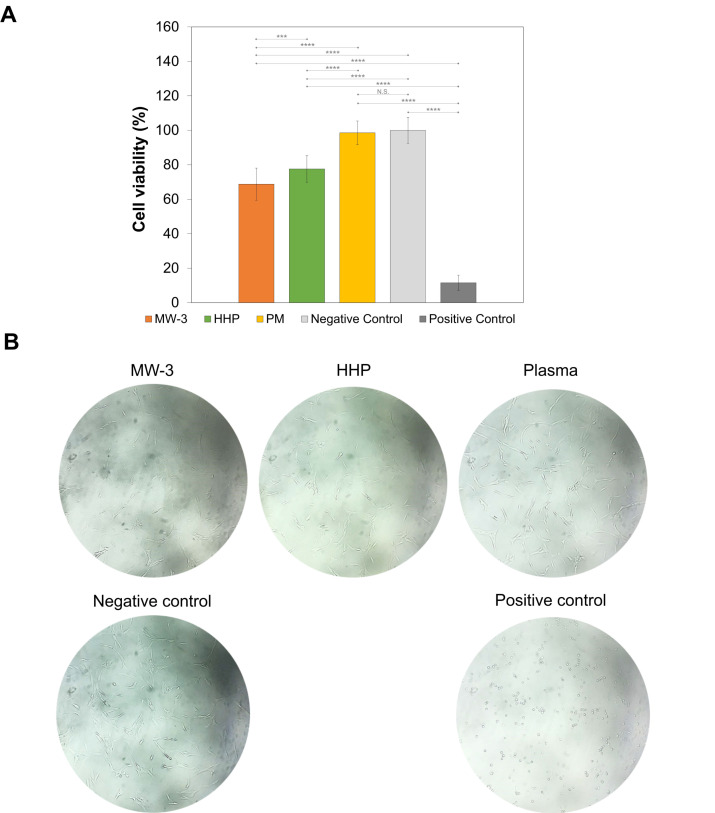
Chondrocyte cell viability (%) determined by the MTT assay (**A**). The relative cell viability is presented as a percentage compared to the negative control. The error bars represent the ±standard deviations (*n* = 4). Optical microscopy images of the incubated chondrocytes after 48 h exposure to the hydrogels’ extracts and controls (**B**). Statistical analysis was performed using a Student-*t* or Willcoxon test, with significance set at *** *p* < 0.005, **** *p* < 0.001. N.S. = not significant.

The biocompatibility of the sterile PVA/PBO hydrogels was studied using an MTT assay, after exposure of the chondrocyte cells to the extracts from the samples. The cell viability was not affected by the extracts from PM samples (98.6 ± 6.8%), but MW-3 and HHP hydrogels led to significantly lower viability (68.8 ± 9.3% and 77.6 ± 7.6%, respectively) (*p* < 0.001) (Figure 12A). According to ISO 10993-5:2009, a material is considered non-cytotoxic when the cellular viability ≥70% [56]. While HHP and PM samples led to values above this threshold, MW-3 was slightly below. However, the sensitivity of this assay was significantly higher than in vivo tests, so it is possible to consider the MW-3 samples as within the limit of non-cytotoxicity.

Figure 12B shows optical microscopy images of the incubated chondrocytes. Although the cell proliferation had decreased for MW-3 and HHP, it can be observed that cells displayed the normal elongated chondrocyte morphology, as in the negative control.

## 3. Conclusions

In this work, the effect of three non-conventional sterilisation methods (i.e., MW, HHP, and PM) on a PVA/PBO hydrogel developed for cartilage replacement was assessed. This hydrogel was able to combine a set of attractive properties that are quite difficult to achieve together in hydrogels, namely a large water content, high mechanical stiffness, low friction, and excellent biocompatibility, which allow mimicking natural cartilage. Since sterilisation is a mandatory step for implantable materials and as hydrogels are generally sensitive materials, finding methods/conditions that ensure the maintenance of hydrogel properties remains a challenge.

The results show that the chosen experimental conditions (MW: 700 W, 3 min; HHP: 600 MPa, 70 °C, 10 min; PM: Ar, 18 W, 5 min) were adequate to ensure sterility in all cases. Sterilisation procedures did not cause significant changes in the surface’ morphology, except for PM, which led to an increase in roughness, due to the etching effect. FTIR analysis showed that the treatments induced some changes in the materials’ chemical structure, in terms of the degree of crystallinity and crosslinking, explaining some differences in its behaviour. The samples’ equilibrium water content did not suffer significant alteration after the treatments, remaining ≈74%. Similarly, the hydrophilicity was also not affected (the water contact angle remained ≈40°). In general, the sterilisation treatments did not negatively impact the materials’ mechanical properties. MW-3 stood out due to an increase in hardness, stiffness (both in tensile and compression), and shear modulus. The coefficient of friction obtained against porcine cartilage, using SSF as lubricant, was significantly lower for these samples, when compared with the non-sterile. HHP led to a similar reduction. HET-CAM tests showed that, after the treatments, the samples remained non-irritant. Moreover, contrarily to PM, MW-3 and HHP led to a decrease in the viability of chondrocytes upon exposure to samples’ extracts. However, the observed values were still above the cytotoxicity threshold.

Overall, among the three studied methods, MW irradiation proved to be the most adequate to sterilise the samples. Due to its high penetration capacity and by achieving high temperatures in the hydrated materials, it is quite efficient, allowing for terminal sterilisation in exposure times as low as 3 min. Furthermore, it led to a general improvement of the mechanical properties of the hydrogel. In turn, argon plasma had some detrimental effects on the material, but more than that, it presents the drawback of not allowing terminal sterilisation, since the materials cannot be processed in their final packaging (due to the low penetration of plasma). Concerning HHP, this led to effective sterilisation of the hydrogel and minor property changes. However, it is surpassed by MW, since it is necessary to have specialized personnel to operate the expensive equipment required.

It must be stressed that in the choice of the ideal sterilisation method of a hydrogel, besides guaranteeing that it keeps its integrity and key properties essential to ensure its functionality, no toxic or hazardous residues should be left in the material. Moreover, other aspects such as a high penetration capability, short processing time, low cost and simplicity and ease of application are also desirable. MW meets all these requisites.

## 4. Materials and Methods

### 4.1. Materials

Polyvinyl alcohol powder (PVA, 99% hydrolysed, average molecular weight 146,000–186,000 g·mol^−1^), phosphate buffer solution (PBS, pH 7.4, buffer strength 150 mM), 3-(4,5-dimethyl-2-thiazolyl)-2,5-diphenyl-2H-tetrazolium bromide (MTT), soybean casein digest broth medium (CASO), Dulbecco’s modified Eagle’s medium (DMEM), calf serum, l-glutamine, penicillin-streptomycin solution (10,000 U·mL^−1^ penicillin, 10 mg·mL^−1^ streptomycin), isopropanol, and dimethyl sulfoxide (DMSO) were all obtained from Sigma-Aldrich (Saint Louis, MO, USA). Poly(p-phenylene-2,6-benzobisoxazole) (PBO, Zylon^®^, AS type) fibres were obtained from Toyobo Co., Ltd. (Osaka, Japan). Thioglycolate liquid medium (TIO) and sodium chloride (purity ≥99%, NaCl) were purchased from PanReac (Barcelona, Spain). Sodium hydroxide (purity ≥99%, NaOH), octylphenoxy-polyethoxyethanol (IGEPAL^®^) were obtained from Merck (Darmstadt, Germany). Trifluoroacetic acid (TFA) (CF_3_COOH) and methanesulfonic acid (99% extra pure MSA, CH_4_O_3_S), were purchased from CARLO ERBA Reagents (Milano, Italy) and ACROS Organics (Thermo Fisher Scientific, Waltham, MA, USA), respectively. Hyaluronic acid sodium salt (HA, average molecular weight of 1,000,000–2,000,000 g·mol^−1^) was purchased from Carbosynth (Compton, Berkshire, UK). Lyophilized bovine serum albumin (BSA, Fraction V, pH 7.0) was provided by Serva Electrophoresis GmbH (Heidelberg, Germany). Human chondrocytes were acquired from CELL Applications, Inc. (San Diego, CA, USA). Special sealed bags (polyamide and polyethylene, 90 μm, 10 × 10 cm^2^) were purchased from Penta Ibérica (Torres Vedras, Portugal). Distilled and deionized (DD) water (18 MΩ cm, pH 7.7) was obtained with a Millipore water purifying system (Millipore system, Millipore Merck, Darmstadt, Germany). Simulated synovial fluid was formulated with 3 mg·mL^−1^ of HA and 4 mg·mL^−1^ of BSA, dissolved in PBS, and stored in a refrigerator at 4 °C between each use.

### 4.2. Synthesis of the Polymeric Materials

PVA hydrogels reinforced with PBO fibres were synthesized according to a protocol described previously [20,57]. Briefly, PVA (6% *w*/*v*) was dissolved in pure TFA, while PBO fibres (1% *w*/*v*) were added to a mixture of 80% TFA and 20% MSA solution. Both solutions were left under magnetic stirring until complete dissolution of the solutes. After complete dissolution, the vials were placed at 45 °C for 30 min, to decrease their viscosity, before mixing. The composites were produced by mixing adequate volumes of PVA and PBO solutions, to obtain a formulation with 6:1 PVA:PBO mass ratio. The PVA/PBO solution was then poured into borosilicate petri dishes and, the lid was put on and left for 1 h. Then a small airflow was let into the dishes for 2 h, by opening the lid slightly. Following this, the lid was completely removed, and the gel was left for an additional 21 h. After complete gelation, the hydrogels were removed from the petri dishes and washed in DD water at 50 °C (7 days, changed 2 times a day, stirring at 200 rpm stirring) to remove acidic components (until the pH was neutral). The washed samples were further cross-linked using a thermal process, in an oven at 45 °C for 48 h. All the samples produced had an average thickness of 2.0 ± 0.5 mm in their hydrated state (unless otherwise indicated).

### 4.3. Sterilisation Processes

#### 4.3.1. Microwave Irradiation (MW)

PVA/PBO hydrogels were individually placed inside special sealed bags, with 3 mL of DD water per 6 g of sample. The sterilisation procedure was carried out using a 2450 MHz microwave oven (Kunft KMW-1698, Worten, Sonae group, Maia, Portugal), at 700 W for 3 min (MW-3 samples) and 5 min (MW-5 samples).

#### 4.3.2. High Hydrostatic Pressure (HHP)

The PVA/PBO samples were packaged for HHP treatment in the same manner as for microwave irradiation. The sealed samples were then submitted to 600 MPa at 70 °C for 10 min in a High Pressure equipment (Hiperbaric 55, Burgos, Spain) (HHP samples) according to [25].

#### 4.3.3. Plasma (PM)

Plasma treatment was carried out using a compact Harrick PDC-32G Plasma Cleaner/Steriliser (115 V, Harrick Plasma, Ithaca, NY, USA). The equipment was connected to a vacuum pump (LVO 100, Leybold, Cologne, Germany) and an argon gas bottle (20 MPa, Alphagaz™, Air Liquid, Lisbon, Portugal). Dry PVA/PBO samples were directly placed into the quartz glass chamber and exposed to ionized argon at 18 W for 5 min (PM samples). Then the samples were placed in sterilised falcon tubes containing 10 mL of DD water per 20 g of sample, in aseptic conditions.

### 4.4. Bioburden

The microbiological load (bioburden) of the non-sterilised PVA/PBO hydrogels (samples with an 8-mm diameter) was evaluated. Buffered peptone broth with sodium chloride (pH 7.0) was sterilised by steam heat in an autoclave at 10^5^ Pa, 121 °C for 20 min, and the samples were immersed in this medium (50 mL) for 2 h at 180 rpm. Afterwards, a pre-selected quantity of the buffer was removed (25 mL) and filtered through cellulose nitrate filters (pore size of 0.45 μm). The used filters were incubated in Sabouraud dextrose agar plates to test for fungi growth (25 °C, 5 days) and in tryptic soy agar (TSA) plates for bacteria growth in aerobic conditions (30 °C, 5 days). The colonies were visually counted to verify the microbiological load of the hydrogels and the mean of triplicates were calculated for each sample.

### 4.5. Sterility Assessment

The sterility of the treated samples was verified following the Portuguese Pharmacopeia [58] and the European Pharmacopeia 10th Edition [59], using the direct inoculation method. Two culture media, TIO and CASO for bacterial and fungi growth, respectively, were prepared and sterilised according to the manufacturer specifications (autoclave, at 10^5^ Pa, 121 °C, for 20 min). Sterilised PVA/PBO samples (8-mm diameter) were placed into the culture medium in a laminar flow cabinet, to ensure aseptic conditions. Two positive controls were performed with bacteria contamination by inoculation of Pseudomonas aeruginosa (ATCC 15442) and Staphylococcus aureus (ATCC 6538) into TIO medium, and a positive control for fungal contamination was carried out by inoculation of the CASO medium with Candida albicans (ATCC 10231). Negative controls (sterile media) were also performed. All flasks were incubated in aerobic conditions for 14 days, at 35 °C (TIO medium) and at 25 °C (CASO medium). Validation of the sterility was performed if no microorganism growth was verified after that period in both media. Triplicates were carried out for each condition.

### 4.6. Characterisation of the Sterile and Non-Sterile PVA/PBO Hydrogels

#### 4.6.1. Morphology

Surface morphology of the non-sterile and sterile samples was analysed through scanning electron microscopy (SEM, Hitachi S2400, Chiyoda, Tokyo, Japan). Samples (squares with 3 × 3 mm^2^) were first dried at 45 °C for 48 h and coated with Au/Pd (100 nm) using a sputter coater and evaporator (Polaron Quorum Technologies, Laughton, East Sussex, UK) for conductivity purposes.

#### 4.6.2. Chemical Structure

The materials’ chemical structure was studied through Fourier transform infrared spectroscopy (FTIR), with attenuated total reflectance (ATR). FTIR equipment (model Spectrum Two from PerkinElmer, Waltham, MA, USA) with a lithium tantalate (LiTaO_3_) mid-infrared (MIR) detector (signal/noise ratio 9300:1) was used. This was equipped with a diamond crystal ATR accessory (model UATR Two). The applied force was controlled, to ensure a good contact between the crystal and samples. Spectra were collected at 4 cm^−1^ resolution and 8 scans of data accumulation and normalized using the OriginPro 8.5 software. Three hydrogel disks (with 14 mm of diameter), non-sterilised and sterilised in the different conditions, were analysed.

#### 4.6.3. Water Content

The non-sterile and sterile samples (6 mm diameter) were dried at 45 °C for 2 days prior to being weighed (dry weight, *W_d_*). Then, they were individually placed in 5 mL of DD water, incubated at 37 °C, and periodically removed and weighed (wet weight, *W_w_*) until a constant value was achieved. The samples were carefully blotted with absorbent paper before each measurement, to remove any remaining water present on their surface. The water content (*WC*) was calculated along the tested time range (120 h), through:

(1) $$WC\;\left(\%\right)=\frac{W_{w}-W_{d}}{W_{w}}\times 100$$

Measurements were performed in triplicate for each condition.

#### 4.6.4. Wettability

The water contact angle on non-sterile and sterilised samples was determined through the captive bubble method (at least one week after treatment). The hydrated hydrogels were fixed to a support and placed inside a quartz glass liquid cell filled with DD water. Air bubbles were formed under the hydrogels’ surface using a syringe with an end curved needle. Pictures were taken at predetermined times for 30 s after bubble deposition using a video camera (jAi CV-A50, Spain), connected to an optical microscope (Wild M3Z, Leica Microsystems, Wetzlar, Germany). Images were analysed using the ADSA software (Applied Surface Thermodynamics Research Associates, Toronto, Canada). At least 10 bubbles were captures in 3 different samples for each condition.

#### 4.6.5. Compressive and Tensile Behaviour

The hydrogels’ compressive and tensile mechanical behaviour was analysed in a TA.XT Express Texture Analyser (Stable Micro Systems, Godalming, UK) with a load cell of 49 N, using the software Exponent. Uniaxial compression tests were performed in unconfined mode.

For the compression tests, the hydrated samples (discs with 8 mm diameter and 4 mm thickness) were completely immersed in DD water at room temperature and compressed at a strain rate of 0.1 mm·s^−1^ up to a 40% strain.

Tensile tests were carried out with a force of 49 N at a speed of 0.5 mm·s^−1^ using hydrated dumbbell-shaped hydrogel samples (5 mm width, 2.5 mm gauge width, 8 mm gauge length, total length 18 mm).

The tangent compressive/tensile modulus (*E_T_*) were calculated in the strain range of 5–35%, in 5% increments, and using the finite difference method [32], through the following equation:

(2) $$E_{T}=\frac{\left(\sigma_{\epsilon }+\Delta_{\epsilon }-\sigma_{\epsilon }-\Delta_{\epsilon }\right)}{2\Delta_{\epsilon }}$$

where *σ_ϵ_* + Δ*_ϵ_* and *σ_ϵ_* − Δ*_ϵ_* are the stresses correspondent to the strains *ϵ* + Δ*_ϵ_* and *ϵ* − Δ*_ϵ_*, respectively and Δ*_ϵ_* was 1%. At least, five experiments were carried out for each condition.

#### 4.6.6. Rheological Behaviour

The viscoelastic properties of the hydrogels were studied using a rheometer MCR 92 (Anton Paar, Ashland, VA, USA) with a parallel plate set up (PP25 geometry) and RheoCompass^TM^ software, Ashland, VA, USA, at 37 °C. Hydrated hydrogel samples (25 mm diameter) were placed at the measurement gap, 15 min prior to the measurements, with DD water surrounding the samples, to avoid evaporation artefacts. Amplitude sweep tests were carried out at a fixed angular frequency (1 Hz) in a shear strain range of 0.01–70%, to determine the plateau values of the material’s storage (*G′*) and loss (*G″*) modulus corresponding to the linear viscoelastic range (LVER). Dynamic frequency sweeps, between 0.1 and 100 rad·s^−1^ were performed, with a strain value fixed within the obtained LVER. Furthermore, the samples were also submitted to an isothermal test for 1 h, at a constant strain of 0.1% (within the LVER) and angular frequency 1 Hz. Triplicates of non-sterile and sterile samples were analysed.

The steady shear rate viscosity of the SSF used in the tribology tests was also measured, for a shear rate range of 0–100 s^−1^ at room temperature. This measurement was performed using a cone-plate geometry (CP50).

#### 4.6.7. Hardness

A Shore A durometer (PCE-DD-A, PCE Instruments, Southampton, UK) was used to measure the hardness of the non-sterile and sterile hydrogels in their hydrated state (DD water). The samples were produced with a 4-mm thickness and cut into disks with an 8-mm diameter. Triplicates were analysed for each condition.

#### 4.6.8. Tribological Behaviour

The coefficient of friction between the hydrogel samples and natural cartilage was measured at room temperature, using the lubricants DD water and SSF. Cartilage pins were harvested from porcine cartilage, obtained from a local butcher’s shop. Full-depth osteochondral plugs were collected using 6-mm hole punchers at randomly selected sites. The cartilage samples’ edges were carefully cut with a scalpel, to prevent sharp edges, washed in DD water, and stored at −20 °C in PBS solution till 2 h before use. The hydrogel samples were pre-hydrated (24 h) in DD water or SSF and placed in a liquid cell with ~25 mL of those lubricant media. The experiments were carried out in a pin-on-disk tribometer TRB3 (Anton Paar, Ashland, VA, USA), in reciprocal oscillating mode (2500 cycles, amplitude 4 mm, sliding velocity 8 mm·s^−1^). The data were collected as a function of time for 1 h, using the InstrumX 9.0.12 software, with an acquisition frequency of 10 Hz. The samples hydrated in water were tested with applied normal loads of 10, 20, and 30 N, corresponding to a Hertzian contact pressure of 0.354, 0.707, and 1.061 MPa, respectively, while the sterile samples were only tested in SSF and for the highest normal load (i.e., 30 N). A minimum of three replicates were analysed for each condition studied.

### 4.7. Irritability Assay

HET–CAM was carried out, to evaluate if PVA/PBO non-sterile and sterile samples could induce a possible adverse irritation reaction in tissues. Fertilized hen eggs (Sociedade Agrícola da Quinta da Freiria S.A., Roliça, Portugal) were incubated in an egg incubator (Intelligent Incubator 56S, Nanchang Edward Technology Co., Ltd., Nanchang, China) for 8 days at 37.0 ± 0.5 °C with a relative humidity of 60 ± 1%. On the 9th day of incubation, the eggs’ shells were cut at the larger far end (which contains the air chamber) using a rotary saw (Dremel 3000 from Breda, Netherlands) to expose the inner membrane. This membrane was hydrated with a NaCl (0.9%) solution, and the egg was placed in the incubator for 30 min.

Afterwards, the inner membrane was removed to expose the CAM. Each sample type was individually placed on top of the CAM for 5 min. The membranes were analysed, to evaluate the appearance of any signals of irritation (i.e., lysis, haemorrhage, and coagulation), and the irritation score was calculated according to [60], using the following equation:

(3) $$IS=\left(\frac{301-T_{H}}{300}\times 5\right)+\left(\frac{301-T_{L}}{300}\times 7\right)+\left(\frac{301-T_{C}}{300}\times 9\right)$$

where *T_H_*, *T_L_*, and *T_C_* represent the time (in seconds) when the first appearance of haemorrhage, lysis, and coagulation occurs, respectively. *IS* classification was done according to the HET-CAM score, ranging from 0–21. An *IS* score of 0–0.9 signifies no signs of irritation, while between 1–4.9 there are signs of slight irritation, from 5–8.9 a moderate irritation occurred, and from 9–21 the eggs show severe irritation [61]. The assay was carried out in triplicate for each hydrogel type. Two controls, a positive, and a negative were utilized, through the addition to the membrane of NaOH (1 M) and NaCl (0.9%), respectively.

### 4.8. Cell Viability

Cytotoxicity analysis of the sterile samples was performed through cell viability assessment. The assays were carried out using the extract test method, in accordance with ISO 10993-5 (ISO 10993—part 5: tests for in vitro cytotoxicity) [56]. The sterile hydrogels were first placed in DMEM (3 cm^2^·mL^−1^) and left for 24 h at 37 °C, in a humified 5% CO_2_ incubator, to obtain the corresponding extract from the hydrogels’ leachate. Human chondrocyte cells were cultured in DMEM, supplemented with 2 mM l-glutamine, 10% calf serum, and 1% penicillin-streptomycin solution, and then seeded in 24-well plates to a density of 0.5 × 10^5^ cell/well and left to incubate for 24 h at 37 °C, with 5% CO_2_ humidity. After that, the cells were inspected using an inverted light optical microscope (Axiovert^®^ 25, ZEISS Microscopy, Jena, Germany), and the culture medium was removed and replaced by the hydrogels’ extract in the same amount. Both positive (DMEM + 10% DMSO) and negative (DMEM) controls were prepared simultaneously. An additional incubation of 48 h was carried out in the same conditions. The cells were visualized using an optical microscope, and micrographs were taken.

Quantification of the cell viability was done using an MTT assay. Briefly, 300 μL of MTT (0.5 mg·mL^−1^ in serum-free DMEM) were added to each well and incubated for 3 h. Following this time, 450 μL of MTT solvent (4 mM HCl, 0.1% IGEPAL in isopropanol) was added to each well. The formed MTT formazan crystals were dissolved by agitating in an orbital shaker for 15 min. Finally, the absorbance was measured in a microplate reader (AMP Platos R 496, AMEDA, Labordiagnostik, Graz, Austria), and the relative quantification of cell viability was normalized to the negative control.

### 4.9. Statistical Analysis

Statistical analysis was carried out, to evaluate the significance of the obtained quantitative data, using the software R Project v. 4.2.1. The normality of the data was verified through the application of a Shapiro–Wilk test. Data with normality were evaluated in terms of similarity of variances with Levene’s test, and subsequently, the data’ significance was evaluated using the parametric tests, one-way ANOVA test and Student’s *t*-test. For the cases where equality of variances did not occur, Welch’s *t*-test was carried out. The non-parametric data was evaluated through the application of the Kruskal–Wallis or Willcoxon tests. The level of significance was set to 0.05.

## Figures and Tables

**Table 1:** Peak intensity ratios of relevant peaks from Figure 3.

Sample	Peak Ratio I_1141_ cm^−1^/I_1085_ cm^−1^	Peak Ratio I_3280_ cm^−1^/I_1085_ cm^−1^	Peak Ratio I_1056_ cm^−1^/I_1085_ cm^−1^
NS	0.635	1.33	1.14
MW-3	0.673	1.35	1.20
HHP	0.675	1.37	1.08
PM	0.662	1.13	1.00

## Data Availability

Raw data are available upon request.
